# Prospective monitoring of patients undergoing radiotherapy during COVID-19

**DOI:** 10.1590/1806-9282.20231421

**Published:** 2024-07-19

**Authors:** Denise Ferreira Silva Alves, Daniela Dornelles Rosa, Luciane Borelli Finatto, Brenda Rigatti, Pedro Tofani Sant' Anna

**Affiliations:** 1 Hospital Moinhos de Vento, Department of Radiation Oncology – Porto Alegre (RS), Brazil.; 2 Hospital Moinhos de Vento – Porto Alegre (RS), Brazil.; 3 Hospital Moinhos de Vento, Department of Radiation Therapy – Porto Alegre (RS), Brazil.

**Keywords:** COVID-19, SARS-CoV-2, Quality of life, Radiotherapy, Therapeutics

## Abstract

**OBJECTIVE:**

The objective of this study was to evaluate the quality of life of consecutive patients undergoing radiotherapy during the coronavirus disease 2019 pandemic at a private hospital in Southern Brazil from September 2020 to September 2021.

**METHODS:**

This study was approved by the Research Ethics Board under project number 112 on April 17, 2020, and it was a prospective descriptive cohort study conducted in a Brazilian radiotherapy department from September 2020 to September 2021. It involved the weekly administration of the European Organisation for Research and Treatment of Cancer Questionnaire Core 30 questionnaires via telephone to consecutively assess patients with pathology-proven cancer diagnoses. These questionnaires captured both demographic data and patients' concerns related to the pandemic, providing a comprehensive overview of their quality of life during radiotherapy treatment.

**RESULTS:**

In this study, 141 patients were analyzed, predominantly female (69.5%) with an average age of 61 years. Breast and prostate were the most treated sites, accounting for 51 and 19% of cases, respectively. The majority of treatments lasted between 3 and 5 weeks (73.77%). A small fraction (4.26%) tested positive for coronavirus disease 2019. The findings also highlighted a relatively high quality of life, with mean global scores of 77.95 and emotional functioning scores of 87.53, indicating maintained well-being during treatment.

**CONCLUSIONS:**

Oncological patients continuing radiotherapy at our center during the pandemic experienced a low coronavirus disease 2019 infection rate and maintained a high quality of life with minimal emotional distress throughout their treatment period.

## INTRODUCTION

In December 2019, the emergence of atypical pneumonia in Wuhan, Hubei Province, China, led to the identification of the causative agent, severe acute respiratory syndrome coronavirus 2 (SARS-CoV-2), by the World Health Organization (WHO). This virus quickly escalated into a global health crisis, with the WHO declaring it an international public health emergency on January 30, 2020, and a pandemic by March 11, 2020^1,2^.

The elderly and those with comorbidities, notably cancer patients, were found to be at increased risk of severe outcomes due to immunosuppression from the disease and treatments. Notably, a study of 2007 coronavirus disease 2019 ­(COVID-19) cases across China indicated a higher incidence among cancer patients (0.9%) compared with the general population (0.29%)^3^. Further research highlighted variable infection rates among different cancer types, with lung cancer showing the highest COVID-19 incidence^4-6^. Another significant finding was the elevated risk of severe infection in cancer patients ­compared with the non-cancer population^3,7^.

Few studies have specifically addressed the impact of COVID-19 on patients undergoing radiotherapy (RT), with one indicating no significant rise in severe events^8^. Despite the global shift toward social isolation or "Lockdown" to curb virus spread, cancer patients continued their treatments, thereby ­possibly heightening their exposure risk.

The dual threat of cancer and COVID-19 significantly affected these patients' fears, compounded by the challenges of isolation, information scarcity, and financial strains ­associated with ongoing cancer care. Amid such unprecedented times, the resultant impact on their quality of life (QoL) underscores the necessity of integrating patient experiences into clinical research. Yet, literature focusing on the QoL among RT patients during the pandemic remains sparse, highlighting the need for focused studies to understand the unique challenges faced by this demographic^9^.

This study aimed to assess the QoL of consecutive patients receiving RT during the COVID-19 pandemic at a private ­hospital in Southern Brazil, from September 2020 to September 2021. It seeks to gain insights into how cancer treatment intersects with pandemic-induced isolation, affecting patient well-being.

## METHODS

We conducted a Research Ethics Board-approved ­investigation within a single Brazilian RT department, prospectively ­assessing consecutive patients with pathologically proven cancer ­diagnoses treated from September 2020 to September 2021. This ­timeframe delineates the period for follow-up and data collection ­activities. The study was approved by the local ethics committee (under project number 112 on April 17, 2020) and ensured that all participants provided informed consent. We assessed all ­consecutive cancer patients treated with RT in our ­department from September 2020 to September 2021. Patients with a ­pathologically proven diagnosis of cancer receiving RT treatment at any site during the study period were included. Patients who were unable to complete the questionnaires, ­including those who were illiterate, were excluded from the study.

This prospective descriptive cohort study involved the weekly administration of the European Organisation for Research and Treatment of Cancer (EORTC) QoL ­questionnaires [Questionnaire Core 30 (QLQ-C30)], incorporating ­demographic data and ­pandemic-related concerns through telephone ­communication. This approach allowed for a ­comprehensive evaluation of patient well-­being during their RT treatment amid the COVID-19 pandemic.

The EORTC QLQ-C30 consists of 30 items that assess five functions (physical, functional, emotional, cognitive, and social), nine symptom subscales/items (fatigue, nausea/­vomiting, pain, dyspnea, insomnia, loss of appetite, ­constipation, ­diarrhea, and financial difficulties), and subscale overall health/QoL^9^. The questionnaire uses a 4-point Likert scale, ­ranging from "not at all" to "a lot," for items 1–28, and a 7-point scale for items 29 and 30, ranging from 1 (very poor) to 7 (­excellent). Scores are linearly transformed on a scale from 0 to 100. A higher score on the functional scale and on global QoL ­indicates ­better ­functioning, while a higher score on the symptom scales ­indicates worse functioning^10,11^.

Patient recruitment, exposure to treatment, and baseline data collection on QoL and demographics occurred from September 2020 to September 2021. No follow-up was required, as the study focused solely on collecting baseline QoL information and ­demographic data, without endpoints such as survival or local control.

This study utilized convenience sampling by including consecutive patients undergoing RT treatment in our department. Given the descriptive nature of this research, calculating a precise sample size was not deemed necessary, as the primary objective was not to test a specific hypothesis but rather to provide a comprehensive overview of the QoL and demographic profiles of our patient cohort during the study period.

## STATISTICAL ANALYSIS

To characterize the study population, we employed descriptive analysis. Categorical variables were summarized using absolute frequencies (n) and percentages (%), and continuous variables were presented as means±standard deviation (SD) or medians with interquartile ranges (IQR: Q1–Q3), depending on data distribution. The Shapiro-Wilk test was utilized to assess data asymmetry.

Quality of life scores were derived according to the ­guidelines in the EORTC QLQ-C30 Scoring Manual. The EORTC QLQ-C30, which is an internationally recognized tool for assessing the health-related QoL of cancer patients, includes five ­functional scales, three symptom scales, and a global health status, all scored from 0 to 100, with higher scores indicating better functioning or more severe symptoms. For this study, we used version 3.0 of the QLQ-C30^11^. Scoring followed a two-step process:

Calculation of the raw score as the average of items ­contributing to the scale: Raw Score (RS)=∑i=1nIinRaw Score (RS)=*n*∑*i*=1*nIi*

1. Linear transformation of the raw score to a 0–100 scale:
2. For functional scales and global health status/QoL: S=(1−RS−1range)×100S=(1−rangeRS−1)×100

3. For symptom scales/items: S=(RS−1range)×100S=(rangeRS−1)×100

The "range" represents the difference between the ­maximum and minimum possible RS values, typically 3 for most items (scored 1–4), except for global health status/QoL items (scored on a 7-point scale, range=6). Our presentation focused on ­specific scales identified by the two authors as most ­representative. Any conflicts were resolved by ­consulting a third, expert author. Scoring procedures are detailed in the EORTC QLQ-C30 Scoring Manual, which provides ­coding for the statistical analysis^11^. Statistical analysis was performed using the SAS software (Statistical Analysis System, SAS Institute Inc., Cary, NC), version 9.4.

## RESULTS

A total of 150 patients were consecutively evaluated for ­inclusion in the study and provided informed consent. However, nine were subsequently excluded for several reasons. Unfortunately, one patient, diagnosed with glioblastoma, died before ­completing the treatment. The other eight patients were excluded due to a lack of response to follow-up calls or their decision to withdraw from the study after initially agreeing to participate.

Among the 141 patients who participated in the study, a ­significant majority (69.5%) were women, with an average age of 61 years (SD±12.9). The predominant site of treatment was the breast (51.1%), followed by the prostate (19.9%). Consistent with global guidelines^12-14^ advocating for ­hypofractionation when feasible, the vast majority of treatments (73.8%) were completed within 3–5 weeks. Notably, a small fraction of the cohort, ­representing 4.3% (6/141), tested positive for COVID-19 during the course of their treatment. Regarding ­transportation methods to the ­hospital, 73.4% of the patients opted for ­individual means, either driving themselves or ­walking. Furthermore, 57.9% of the study ­participants resided alone or with just one other person (Table 1).

**Table 1:** Demographic and treatment characteristics of radiotherapy patients: a snapshot of gender distribution, age, treatment sites, duration, and coronavirus disease 2019 infection rates.

Characteristics		n (%) or mean (±SD)
Gender		
	Female	98 (69.5)
	Male	43 (30.5)
Age (years)		61.2 (12.9)
Site of treatment		
	Breast	72 (51.1)
	Prostate	28 (19.9)
	Gynecological	10 (7.1)
	Gastrointestinal	4 (2.8)
	Thorax	2 (1.4)
	Others	25 (17.7)
Duration of treatment (weeks)		
	1	7 (5,0)
	2	10 (7.1)
	3	31 (22.0)
	4	29 (20.6)
	5	44 (31.2)
	6	15 (10.6)
	7	5 (3)
Means of transport to the hospital		
	Public	37 (26.2)
	Individual	93 (66.0)
	Walking	11 (7.8)
Number of persons with whom the patient lives		
	0	16 (11.4)
	1	65 (46.4)
	2	33 (23.6)
	3	21 (15.0)
	4	5 (3.6)
Positive test for COVID-19		6 (4.3)
	During treatment	3 (2.1)
	Right after	3 (2.1)

Demographic and treatment characteristics of 141 patients who underwent radiotherapy were included in our analysis.

In Table 2, we present patients' concerns during RT, ­showing varied levels of insecurity about treatment, with 46.8% ­reporting less concern. The majority (99.3%) felt no need for additional information about COVID-19. Confidence in the hospital's precautionary measures was high, with 78.0% expressing a great sense of security.

**Table 2:** Patient concerns and perceptions during radiotherapy in the coronavirus disease 2019 pandemic: levels of insecurity, information sufficiency, and trust in hospital safety protocols.

Insecurity about the treatment during the pandemic (1—little to 7—a lot)		n (%) or mean
	1	66 (46.8)
	2	6 (4.3)
	3	10 (7.1)
	4	9 (6.4)
	5	18 (12.8)
	6	6 (4.3)
	7	26 (18.4)
Need to receive more information about COVID-19 during the treatment		
	No	140 (99.3)
	Yes	1 (0.7)
Sense of security with the precautionary protocol instituted in the hospital (1—little to 7—a lot)		
	1	5 (3.6)
	2	1 (0.7)
	3	1 (0.7)
	4	2 (1.4)
	5	7 (5.0)
	6	15 (10.6)
	7	110 (78.0)

This table reveals patients' responses regarding their concerns and perceptions during radiotherapy amid the COVID-19 pandemic. A significant portion (46.8%) reported less insecurity about treatment, while a substantial majority (99.3%) felt no need for additional COVID-19 information. Confidence in the hospital's precautionary measures was overwhelmingly high, with 78.0% expressing a great sense of security.

Table 3 shows a consistent QoL (EORTC QLQ-C30) and functional status among patients over the treatment period, with median global health status/QoL scores remaining stable across the initial weeks and slightly decreasing by the seventh week. Physical, role, emotional, cognitive, and social ­functioning scores were generally high, reflecting minimal impact on patients' overall well-being. The EORTC QLQ-C30 revealed a mean global QoL score of 77.9 and an emotional functioning score of 87.5, indicating that patients sustained high QoL and minimal emotional distress during the study period. Symptom subscale analysis highlighted fatigue, insomnia, pain, and ­appetite loss as the most significant issues, with scores ranging from 19.7 (fatigue) to 10.3 (appetite loss). Dyspnea, ­potentially ­indicative of COVID-19, scored the lowest at 3.4, aligning with the observed low infection rates within the sample.

**Table 3:** Evolution of patient functional well-being over time: a week-by-week analysis of quality of life and functional scales during radiotherapy treatment.

		Week 1 (n=141)	Week 2 (n=134)	Week 3 (n=123)	Week 4 (n=94)	Week 5 (n=64)	Week 6 (n=21)	Week 7 (n=7)	Mean (±SD)
Global health status/QoL		83 (67–92)	83 (67–92)	83 (67–92)	83 (67–92)	83 (67–83)	83 (67–83)	67 (33–83)	77.9 (18.5)
Functional scales									
	Physical functioning	93 (73–100)	93 (80–100)	93 (80–100)	93 (80–100)	93 (80–100)	100 (90–100)	100 (100–100)	88.3 (16.8)
	Role functioning	100 (83–100)	100 (83–100)	100 (83–100)	100 (67–100)	100 (100–100)	100 (100–100)	100 (100–100)	88.1 (25.1)
	Emotional functioning	92 (75–100)	92 (83–100)	92 (83–100)	92 (75–100)	100 (79–100)	100 (92–100)	100 (100–100)	87.5 (19.6)
	Cognitive functioning	100 (83–100)	100 (83–100)	100 (83–100)	100 (83–100)	100 (83–100)	100 (83–100)	100 (83–100)	89.6 (17.6)
	Social functioning	100 (67–100)	100 (83–100)	100 (67–100)	100 (67–100)	100 (67–100)	100 (67–100)	100 (100–100)	84.7 (31.2)

This table provides a comprehensive overview of the changes in patients' general functions over the course of their radiotherapy treatment, spanning from the first to the seventh week. It also documents median and mean scores for global health status/quality of life (QoL), along with functional scales, including physical, role, emotional, cognitive, and social functioning.

Approximately 10–15 days post-treatment, a final survey was conducted, comprising questions about QoL, general health, and symptoms specifically related to the COVID-19 infection such as any kind of breathing difficulty, cough, sore throat, and fever. Some patients with typical symptoms of COVID-19 were instructed to perform the test, and the results revealed that three of them tested positive for the virus.

## DISCUSSION

This study provides a comprehensive look at the ­experiences of patients receiving RT during the COVID-19 pandemic. It reveals a complex response marked by initial concern and strong trust in hospital protocols, evidenced by 78% of patients expressing confidence, leading to stable or improved well-­being. Despite fears of increased COVID-19 risk for ­cancer patients noted in other studies^3,4,6,7^, our cohort showed low ­infection rates (5%), suggesting effective preventive measures. Contrary to expectations of heightened emotional distress^15^, our ­findings indicate maintained QoL, underscoring the value of ­personalized care and robust safety protocols in ensuring patient well-being during challenging times.

In exploring the impact of COVID-19 on cancer patients' QoL, Ciążynska et al. identified significant declines in global QoL, particularly in cognitive and social functioning, while maintaining near-normal levels in physical and emotional aspects^16^. This contrasted with our findings, which did not ­investigate a population receiving RT in a department ­characterized by well-organized processes like ours. This ­difference in study settings may account for the variations in outcomes observed between the two studies.

Our study's low COVID-19 infection rates among RT patients contrast with broader data, indicating a heightened risk for cancer patients. Liang et al.^3^ and Dai et al.^4^ reported increased infection and serious complication rates in ­cancer patients, particularly those with lung, gastrointestinal, and breast cancers^5-7^. Despite the higher risk in general ­cancer ­populations, our focused study in a well-organized RT ­department showed a notably low ­infection rate of 4.2%. This suggests that rigorous hygiene protocols, team training, patient education, and enforced social distancing may have effectively ­mitigated the risk of COVID-19 transmission within this ­specific ­treatment setting.

While studies on RT patients during the pandemic are scarce, one notable finding is the lack of a significant increase in serious events, despite the high patient turnover in a closed treatment environment. Our study reported a low ­COVID-19 infection rate of 4.2%, predominantly among patients receiving adjuvant treatment for breast tumors. Although it is challenging to definitively attribute this low rate to the stringent hygiene protocols, staff training, patient education, and enforced social isolation, these measures likely played a key role. Contrary to reports of emotional distress among ­quarantined individuals, our findings indicate maintained QoL and emotional ­functioning among our cohort, suggesting effective ­management of patient well-being during treatment.

Alongside personalized approaches such as weekly phone calls and daily screenings, the precautionary measures implemented to curb infection and enhance patient care during the pandemic likely played a pivotal role in our positive outcomes. These strategies suggest that patients felt comprehensively supported at our center during the COVID-19 crisis. This experience invites a broader contemplation on reorganizing healthcare services toward more personalized, patient-centric models, emphasizing the importance of humanized medicine in future healthcare delivery.

Finally, our study findings are significant yet underscored by limitations that warrant a nuanced understanding. Conducted at a single institution, the scope of this study may limit the ­generalizability of its results. The small sample size and ­limited participant number restrict our ability to perform robust ­statistical analyses and generalize findings. Moreover, the absence of ­baseline data before and during the COVID-19 pandemic, along with the use of the EORTC QLQ-C30—a globally ­validated tool, raises questions about its adequacy in fully capturing the pandemic's impact on patients' emotional and psychological well-being. Future research could ­benefit from developing tools specifically aimed at assessing the ­emotional well-being of ­cancer patients in pandemic ­scenarios, ­potentially offering more targeted and reliable insights.

## CONCLUSION

During the COVID-19 pandemic, our study found that cancer patients undergoing RT maintained a high QoL with ­minimal emotional distress, alongside a low COVID-19 ­infection rate. These results highlight the effectiveness and safety of ­continuing RT treatments during such crises. The limited cases of COVID-19 in our cohort restrict a detailed analysis of long-term COVID implications. Nonetheless, our findings provide insights for managing oncological care in future ­pandemics, ­emphasizing the resilience and adaptability of healthcare ­delivery in ­challenging times.

## ETHICS APPROVAL AND CONSENT TO PARTICIPATE

This study adhered to the principles outlined in the Helsinki Declaration. This study was approved by the Hospital Moinhos de Vento Research Ethics Committee under REDCap no. 112 on April 17, 2020, and authorization was granted to use the EORTC QLQ-30 questionnaire. Patients were enrolled from September 2020 to September 2021, and informed consent was obtained at the time of recruitment.

## Data Availability

All data generated or analyzed during this study are included in this published article.
